# Dendrobine Attenuates Cisplatin-Induced Acute Kidney Injury with KEAP1–NRF2 Activation and Reduced Ferroptosis-Associated Stress

**DOI:** 10.3390/antiox15101337

**Published:** 2026-10-16

**Authors:** Yunbo Yan, Songmei Hu, Mingxin Chen, Yanlin Wen, Xinyu He, Aoli Ding, Chengyuan Tang, Junxiang Chen

**Affiliations:** 1 Department of Nephrology, The Second Xiangya Hospital of Central South University, Changsha 410011, China; yanyunbo@csu.edu.cn (Y.Y.); 248212224@csu.edu.cn (S.H.); 248201018@csu.edu.cn (M.C.); 258211026@csu.edu.cn (X.H.); dingaoli0517@csu.edu.cn (A.D.); 2 Department of Nephrology, Hunan Key Laboratory of Kidney Disease and Blood Purification, The Second Xiangya Hospital of Central South University, Changsha 410011, China; wenyanlinnn@csu.edu.cn; 3 Xiangya School of Nursing, Central South University, No. 172 Tongzipo Road, Yuelu District, Changsha 410013, China

**Keywords:** acute kidney injury, dendrobine, ferroptosis, KEAP1–NRF2, lipid peroxidation

## Abstract

Acute kidney injury (AKI) lacks effective pharmacological therapies, and ferroptosis-associated lipid peroxidation contributes to renal tubular injury. Here, we investigated the protective effects of dendrobine against AKI and its mechanisms. Dendrobine was evaluated in murine cisplatin-induced AKI and renal ischemia–reperfusion (I/R) models and in cisplatin- and erastin-challenged HK-2 cells using functional, histological, biochemical, and molecular analyses. Dendrobine improved renal function, alleviated tubular injury, reduced oxidative stress and lipid peroxidation, and preserved the GPX4/SLC7A11 antioxidant defense system in cisplatin-induced AKI and renal tubular cells. Dendrobine also attenuated erastin-associated lipid peroxidation and glutathione depletion, supporting an effect on ferroptosis-associated redox stress. Consistent renoprotective effects were observed in the renal I/R model. Mechanistically, target-engagement and protein-interaction analyses supported cellular engagement of KEAP1 by dendrobine and showed reduced KEAP1–NRF2 association and NRF2 ubiquitination, accompanied by enhanced NRF2 stabilization and nuclear accumulation. NRF2 inhibition attenuated the protective effects of dendrobine. Moreover, the KEAP1 R415A substitution attenuated dendrobine-associated KEAP1 engagement, NRF2 activation, and cytoprotection, supporting a functional contribution of Arg415 to these responses. Collectively, these findings support KEAP1–NRF2-regulated antioxidant defense as an important component of dendrobine-mediated protection against renal tubular injury and ferroptosis-associated stress in AKI.

## 1. Introduction

Acute kidney injury (AKI) is a common and life-threatening clinical syndrome characterized by an abrupt decline in renal function following nephrotoxic exposure, inflammation, or ischemia–reperfusion injury (IRI) [1,2]. AKI affects approximately 10–15% of hospitalized patients and more than half of critically ill patients and is associated with increased mortality, prolonged hospitalization, and an elevated risk of subsequent chronic kidney disease [3,4]. Cisplatin-induced nephrotoxicity remains a major clinical challenge because renal injury frequently limits the use of this widely administered chemotherapeutic agent [5,6,7]. Despite substantial advances in understanding AKI pathogenesis, effective pharmacological therapies remain limited. Identifying targetable mechanisms that drive renal tubular injury is therefore essential for developing new therapeutic approaches.

Ferroptosis, an iron-dependent form of regulated cell death characterized by excessive lipid peroxidation and impaired antioxidant capacity, has emerged as an important mechanism of renal tubular injury [8,9]. Accumulating evidence implicates ferroptosis as a key contributor to cisplatin-induced AKI, and pharmacological or genetic inhibition of ferroptosis alleviates renal injury in experimental models [10,11,12,13]. However, the upstream mechanisms governing ferroptotic susceptibility in renal tubular epithelial cells remain incompletely understood. Identifying druggable regulators that enhance endogenous antioxidant defenses may therefore offer a strategy to limit ferroptotic renal injury.

The Kelch-like ECH-associated protein 1 (KEAP1)–nuclear factor erythroid 2-related factor 2 (NRF2) pathway is a central regulator of cellular redox homeostasis and ferroptosis resistance [14]. Under basal conditions, KEAP1 promotes NRF2 ubiquitination and proteasomal degradation, whereas disruption of KEAP1-mediated repression stabilizes NRF2 and induces antioxidant and cytoprotective genes, including GPX4 and SLC7A11/xCT [14,15,16]. Recent studies have shown that natural small molecules can modulate this checkpoint in disease models. Luteoloside was reported to disrupt the KEAP1–NRF2 interaction, reduce NRF2 ubiquitination, and suppress ferroptosis in renal ischemia–reperfusion injury [17], whereas kaempferol-3-O-α-L-(4″-E-p-coumaroyl)-rhamnoside (KAE) was reported to act through an Arg415-dependent KEAP1–NRF2/GPX4 mechanism in acute lung injury [18]. These findings highlight KEAP1–NRF2 signaling as a potential therapeutic target for limiting ferroptosis-associated renal injury and support further investigation of natural small molecules as modulators of this pathway in AKI.

Dendrobine, a sesquiterpene alkaloid isolated from Dendrobium nobile, has antioxidant, anti-inflammatory, and neuroprotective activities [19,20,21,22]. Previous studies have implicated NRF2 signaling in its cytoprotective effects [23,24,25,26]. During the preparation and submission of this manuscript, Xiang et al. reported that dendrobine interacts with the KEAP1 Kelch domain, disrupts the KEAP1–NRF2 interaction, and implicates Arg415 in a model of diabetic cardiomyopathy [27]. Here, our study was independently initiated and conducted to investigate the potential effects and underlying mechanisms of dendrobine in AKI. We investigated dendrobine in cisplatin-induced AKI and in cisplatin- and erastin-challenged HK-2 cells, focusing on KEAP1-NRF2 signaling, NRF2-dependent antioxidant defense, and ferroptosis-associated stress. A renal ischemia–reperfusion (I/R) model was additionally used to provide supportive evidence of renoprotective activity. Our findings support a role for KEAP1–NRF2-regulated antioxidant defense in dendrobine-mediated protection against cisplatin-induced AKI and attenuation of ferroptosis-associated stress.

## 2. Materials and Methods

### 2.1. Reagents and Antibodies

A detailed summary of the antibodies used in this study is provided in Supplementary Table S1. Dendrobine (purity ≥ 98%, #D302777) was obtained from Aladdin (Shanghai, China). Erastin (#T1765) was purchased from TargetMol (Shanghai, China). Cisplatin (#HY-17394), ferrostatin-1 (Fer-1, #HY-100579) and ML385(#HY-100523) were purchased from MedChemExpress (Monmouth Junction, NJ, USA), and MG132 (#S2619) was obtained from Selleck Chemicals (Houston, TX, USA). The TUNEL assay kit (#KGA700) was purchased from KeyGEN BioTECH (Nanjing, China). The malondialdehyde (MDA; #S0131S), superoxide dismutase (SOD; #S0101S), and reactive oxygen species (ROS, #S0033S) detection kits were supplied by Beyotime Biotechnology (Shanghai, China). Kits for glutathione (GSH; #A006-2-1), blood urea nitrogen (BUN; #C013-2-1), and serum creatinine (Scr; #C011-2-1) were purchased from Nanjing Jiancheng Bioengineering Institute (Nanjing, China). The iron assay kit (#E-BC-K773-M) was obtained from Elabscience (Wuhan, China). BODIPY™ 581/591 C11 lipid peroxidation sensor (#D3861) and lipo3000 (# L3000075) were purchased from Invitrogen (Thermo Fisher Scientific, Waltham, MA, USA).

### 2.2. Animals and Treatment

Eight-week-old male C57BL/6J mice (22 ± 2 g) were obtained from Hunan SJA Laboratory Animal Co., Ltd. (Changsha, China). All animals were housed under specific pathogen-free (SPF) conditions at 22 ± 2 °C and 55 ± 5% relative humidity, with a 12 h light/12 h dark cycle and free access to food and water. Animal experimental protocols were reviewed and approved by the Animal Ethics Committee of Central South University (Approval No. 20260749), and all procedures were performed in accordance with institutional guidelines for animal care and use.

For cisplatin-induced AKI, mice were randomly allocated to four groups (n = 6 per group): control, dendrobine, cisplatin, and cisplatin plus dendrobine. Cisplatin was administered intraperitoneally at 25 mg/kg as a single dose, whereas dendrobine was administered intraperitoneally at 20 mg/kg once daily based on preliminary experiments and previous studies [19,28]. In the combination group, dendrobine and cisplatin were administered concurrently, followed by continued once-daily administration of dendrobine. Mice were euthanized on day 3 after cisplatin administration, and blood and kidney tissues were collected for subsequent analyses.

For renal I/R-induced AKI, mice were randomly divided into three groups (n = 6 per group): sham, IRI, and IRI + dendrobine. Dendrobine (20 mg/kg) was administered intraperitoneally once daily starting 1 day before surgery. Bilateral renal pedicles were clamped for 30 min to induce ischemia, followed by 24 h of reperfusion. Sham-operated mice underwent the same surgical procedures without renal pedicle clamping. Blood and kidney tissues were collected at the end of the experiments for subsequent analyses.

### 2.3. Renal Function and Histologic Examination

Blood samples were collected and centrifuged at 5000 rpm for 20 min at room temperature to obtain serum. Serum creatinine (Scr) and blood urea nitrogen (BUN) levels were measured using commercial assay kits (Nanjing Jiancheng Bioengineering Institute, Nanjing, China) according to the manufacturer’s instructions. Half of each kidney was rapidly frozen in liquid nitrogen and stored at −80 °C for subsequent biochemical and molecular analyses. The remaining half of each kidney was fixed in 10% formalin, followed by dehydration, paraffin embedding, and sectioning at a thickness of 4 μm. The sections were subjected to hematoxylin and eosin (H&E) and periodic acid–Schiff (PAS) staining for histopathological evaluation. Tubular injury was scored semi-quantitatively based on the percentage of damaged tubules, as described in previous studies [29,30]. The scoring criteria were as follows: 0 = no injury; 1 = injury < 25%; 2 = 25–50% injury; 3 = 50–75% injury; and 4 = injury > 75%.

### 2.4. Immunohistochemistry

Paraffin-embedded kidney sections (4 μm) were deparaffinized, rehydrated, and subjected to antigen retrieval. After blocking with 5% normal goat serum, sections were incubated overnight at 4 °C with primary antibodies against F4/80, GPX4, SLC7A11/xCT, or 4-HNE. Immunohistochemical staining was performed using a commercial polymer-based detection system according to the manufacturer’s instructions, followed by hematoxylin counterstaining. Stained sections were examined under a light microscope at 200× magnification, and the staining intensity or positive area was quantified using ImageJ (version 1.54d).

### 2.5. TUNEL Staining

Renal tubular cell death in tissue sections was assessed using a commercial TUNEL assay kit according to the manufacturer’s instructions. TUNEL-positive cells were quantified in randomly selected microscopic fields.

### 2.6. Cell Culture

HK-2 cells were obtained from the Chinese Academy of Sciences (Shanghai, China) and cultured in DMEM/F12 medium supplemented with 10% fetal bovine serum and 1% penicillin–streptomycin. Cells were maintained at 37 °C in a humidified atmosphere with 5% CO_2_.

### 2.7. Plasmid Construction and Transfection

The human full-length KEAP1 expression plasmid [pCMV-KEAP1(human)] and the corresponding R415A mutant plasmid [pCMV-KEAP1(human)-R415A] were purchased from Miaoling Biology (Wuhan, China). The KEAP1-R415A mutant was generated by substituting arginine (Arg) at position 415 with alanine (Ala). HK-2 cells were seeded in 10-cm culture dishes and, at approximately 50% confluence, transiently transfected with 10 μg of the indicated plasmid DNA per dish using Lipofectamine™ 3000 Transfection Reagent (Invitrogen, Carlsbad, CA, USA) according to the manufacturer’s instructions. Cells were subjected to subsequent treatments 24 h after transfection.

### 2.8. Cell Viability Assay

Cell viability was determined using a Cell Counting Kit-8 (CCK-8) assay according to the manufacturer’s instructions. HK-2 cells were seeded into 96-well plates and cultured overnight. Where indicated, cells were pretreated with pharmacological inhibitors for 1 h, followed by dendrobine pretreatment for an additional 1 h before exposure to the indicated stimulants, including cisplatin or erastin, for the designated time periods. Cells treated with dendrobine alone received dendrobine for the indicated duration. After treatment, the CCK-8 reagent was added to each well and incubated according to the manufacturer’s instructions. Absorbance at 450 nm was measured using a microplate reader. Specific drug concentrations and treatment durations are provided in the corresponding figure legend.

### 2.9. Western Blotting

Proteins were resolved by SDS-PAGE (8–12.5%) and transferred onto PVDF membranes. After blocking, membranes were incubated with primary antibodies overnight at 4 °C, followed by incubation with corresponding secondary antibodies at room temperature for 1 h. Protein bands were visualized using an enhanced chemiluminescence detection system.

### 2.10. Co-Immunoprecipitation and Ubiquitination Assays

HK-2 cells were treated with cisplatin (30 μM) alone or in combination with dendrobine for 18 h. MG132 (10 μM) was added only for ubiquitination assays, followed by an additional 6 h incubation, whereas no MG132 was used for Co-IP experiments. For Co-IP, cells were lysed in IP lysis buffer supplemented with protease inhibitors, whereas RIPA buffer was used for ubiquitination assays. The anti-NRF2 antibody was incubated with Protein A/G magnetic beads at 4 °C for 3 h, followed by incubation with cell lysates overnight. After washing with ice-cold lysis buffer, the beads were boiled in SDS sample loading buffer, and the eluted proteins were analyzed by Western blotting to assess NRF2 interactions and ubiquitination levels.

### 2.11. Biochemical and Oxidative Stress Assays

Renal MDA, GSH, SOD, and serum Fe^2+^ levels were measured using commercial assay kits according to the manufacturers’ instructions. Intracellular ROS levels were assessed using DCFH-DA staining. Briefly, HK-2 cells were incubated with DCFH-DA at 37 °C for 30 min, washed with PBS, and examined under a fluorescence microscope. ROS fluorescence intensity was quantified using ImageJ. Mitochondrial membrane potential (Δψm) was evaluated using JC-1 staining according to the manufacturer’s instructions, and the red-to-green fluorescence intensity ratio was used to indicate changes in Δψm. Lipid peroxidation was assessed using BODIPY™ 581/591 C11. Briefly, HK-2 cells were incubated with 10 μM BODIPY™ 581/591 C11 at 37 °C for 30 min in the dark, followed by PBS washing and fluorescence imaging. The oxidation-dependent fluorescence shift was quantified using ImageJ.

### 2.12. Molecular Docking

The crystal structures of the KEAP1 BTB domain (PDB ID: 4CXI) and Kelch domain (PDB ID: 4L7B) were obtained from the Protein Data Bank [31,32]. The molecular structure of dendrobine (PubChem CID: 442523) was obtained from the PubChem database. Molecular docking was performed using the CB-Dock2 web server (http://cao.labshare.cn:11080/cb-dock2/php/blinddock.php, accessed on 13 August 2025) [33]. The docking model with the highest predicted affinity was further analyzed using PyMOL (version 3.1.6.1; Schrödinger LLC, New York, NY, USA) and the Protein–Ligand Interaction Profiler (PLIP, https://plip-tool.biotec.tu-dresden.de/plip-web/plip/index, accessed on 16 August 2025) to characterize potential protein–ligand interactions [34].

### 2.13. CETSA and DARTS Assays

Cellular thermal shift assay (CETSA) and drug affinity responsive target stability (DARTS) assays were performed to assess cellular target engagement of KEAP1 by dendrobine, as previously described [35,36]. These stability-based assays can support target engagement but do not provide an equilibrium dissociation constant or establish a direct residue-level contact. For CETSA, HK-2 cells were treated with dendrobine (80 μM) for 4 h, followed by cell lysis. Cell lysates were aliquoted and heated at the indicated temperatures for 3 min, followed by cooling and centrifugation at 16,000 rpm for 10 min at 4 °C. The supernatants were collected and analyzed by Western blotting to assess the thermal stability of KEAP1. For DARTS, cell lysates were incubated with dendrobine (20, 40, 80 μM) or vehicle at 37 °C for 30 min, followed by treatment with pronase E (2.5 μg/mL) at 37 °C for 20 min. Proteolysis was terminated by adding SDS sample loading buffer and heating the samples, followed by Western blot analysis of KEAP1.

### 2.14. Statistical Analysis

Data are presented as the mean ± SD unless otherwise indicated. Statistical analyses were performed using GraphPad Prism 10. Two-group comparisons were evaluated using two-tailed unpaired Student’s *t*-tests. Multiple-group comparisons were evaluated using one-way ANOVA with Tukey’s multiple-comparisons test; experiments with two independent factors were evaluated using two-way ANOVA with Sidak’s multiple-comparisons test. A *p* value < 0.05 was considered statistically significant. Sample sizes and significance thresholds are provided in the corresponding figure legends. The reported n values denote independent biological replicates unless otherwise specified.

## 3. Results

### 3.1. Dendrobine Protects Against Cisplatin-Induced Acute Kidney Injury in Mice

**Figure 1:**
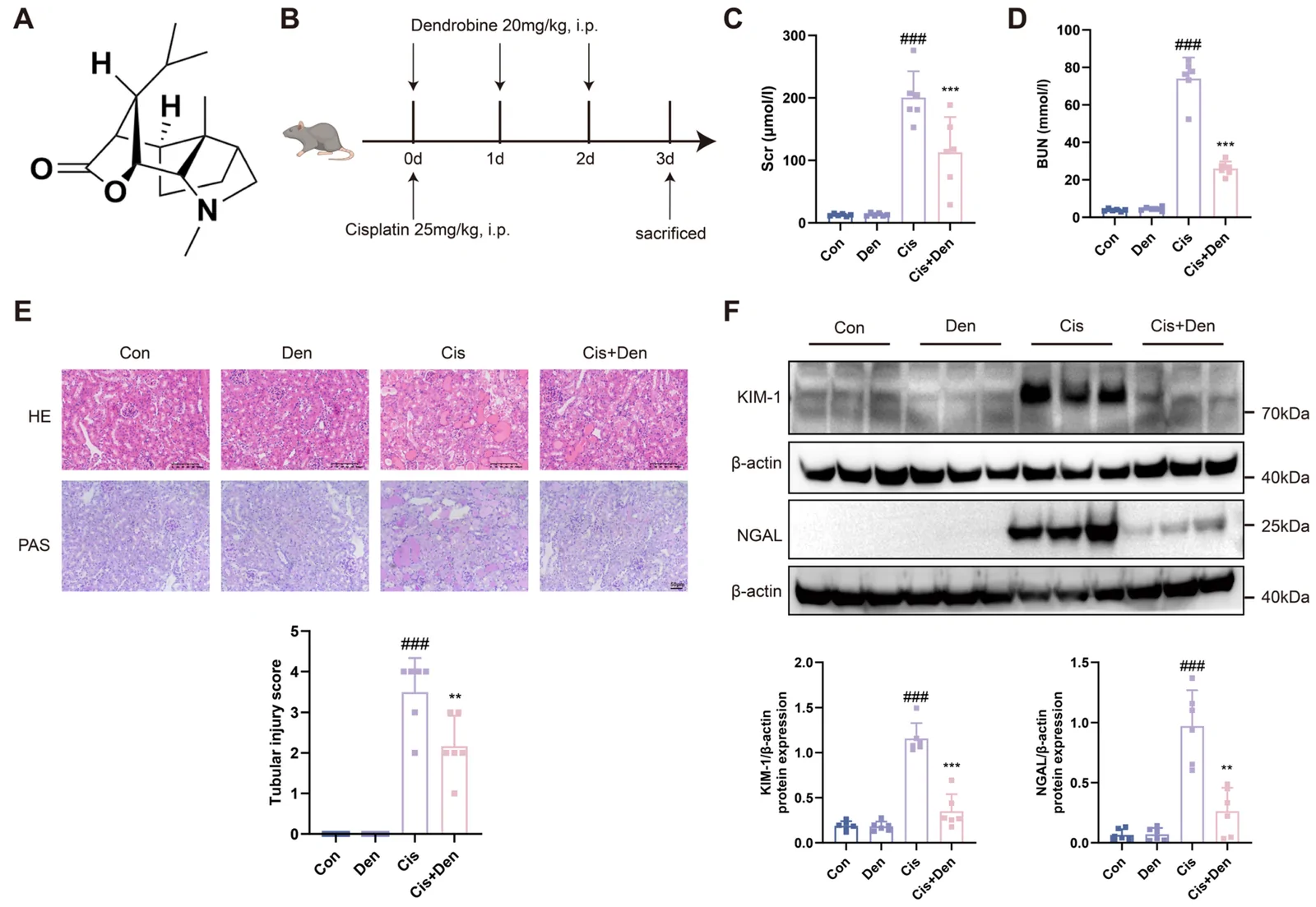
Dendrobine protects mice against cisplatin-induced acute kidney injury. (**A**) Chemical structure of dendrobine. (**B**) Schematic illustration of the experimental design. (**C**,**D**) Serum creatinine (Scr) and blood urea nitrogen (BUN) levels. (**E**) Representative H&E- and PAS-stained kidney sections and renal injury scores. Scale bars, 100 μm for H&E and 50 μm for PAS. (**F**) Representative immunoblots and quantitative analysis of KIM-1 and NGAL protein levels. Protein levels were normalized to β-actin and expressed relative to the corresponding control group. Data are presented as mean ± SD. Each dot represents an individual mouse. ^###^ *p* < 0.001 vs. Con group; ** *p* < 0.01, *** *p* < 0.001 vs. Cis group; n = 6 per group.

To evaluate the renoprotective effect of dendrobine (chemical structure shown in Figure 1A), a cisplatin-induced acute kidney injury (CI-AKI) model was established in C57BL/6J mice by intraperitoneal injection of cisplatin (Figure 1B). As shown in Figure 1C,D, dendrobine significantly improved renal function, as evidenced by reduced serum creatinine and blood urea nitrogen levels. H&E and PAS staining revealed severe tubular injury in the CI-AKI group, characterized by loss of the brush border, tubular dilatation, protein cast formation, and tubular necrosis. These pathological alterations were markedly alleviated following dendrobine treatment (Figure 1E). Consistently, dendrobine significantly decreased the expression of the tubular injury markers KIM-1 and NGAL (Figure 1F), further indicating its protective effect against renal tubular damage. In addition, dendrobine markedly attenuated tubular cell apoptosis, as evidenced by reduced TUNEL-positive staining and decreased cleaved caspase-3 expression (Supplementary Figure S1A–D). Dendrobine also exerted a potent anti-inflammatory effect, as evidenced by reduced F4/80-positive staining (Supplementary Figure S1E,F) and decreased expression of the pro-inflammatory cytokines TNF-α, IL-1β, and IL-6 (Supplementary Figure S1G,H). Collectively, these findings demonstrate that dendrobine effectively protects against cisplatin-induced AKI by preserving renal function and mitigating tubular injury, apoptosis, and inflammation.

### 3.2. Dendrobine Attenuates Ferroptosis-Associated Changes in Cisplatin-Induced AKI In Vivo

**Figure 2:**
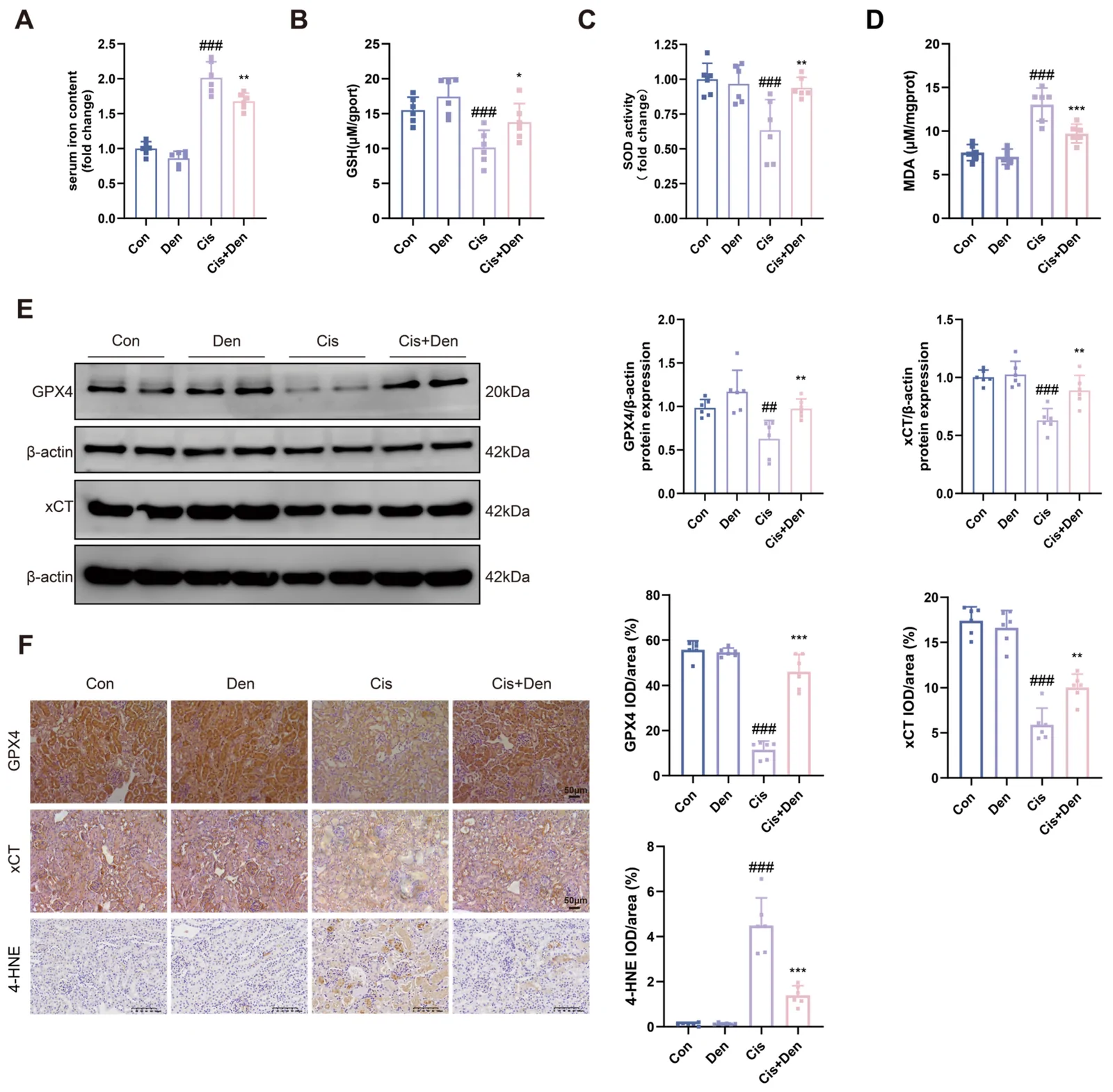
Dendrobine attenuates ferroptosis-associated oxidative stress in cisplatin-induced AKI. (**A**) Serum Fe^2+^ levels. (**B**–**D**) Renal GSH levels, SOD activity, and MDA levels. (**E**) Representative immunoblots and quantitative analysis of GPX4 and xCT protein levels. (**F**) Representative immunohistochemical images and quantitative analysis of GPX4 and xCT expression, and 4-HNE accumulation. Scale bars, 50 μm for GPX4 and xCT and 100 μm for 4-HNE. Protein levels were normalized to β-actin and expressed relative to the corresponding control group. ^##^ *p* < 0.01, ^###^ *p* < 0.001 vs. Con group; * *p* < 0.05, ** *p* < 0.01, *** *p* < 0.001 vs. Cis group; n = 6 per group.

Because ferroptosis-associated stress contributes to cisplatin-induced AKI, we examined whether dendrobine attenuated ferroptosis-associated alterations in vivo. Cisplatin administration increased serum Fe^2+^ levels and renal MDA content, while decreasing renal GSH levels and SOD activity. These findings indicate altered circulating Fe^2+^ levels together with increased renal lipid peroxidation and impaired antioxidant capacity (Figure 2A–D), and these changes were significantly attenuated by dendrobine treatment. We next examined the GPX4/xCT antioxidant defense system, which is central to ferroptosis resistance. Western blotting showed that cisplatin markedly decreased renal GPX4 and xCT protein expression, whereas dendrobine significantly restored both proteins (Figure 2E). Consistently, immunohistochemical analysis revealed reduced GPX4 and xCT staining in the kidneys of cisplatin-treated mice, which was partially restored by dendrobine. In contrast, renal accumulation of 4-hydroxynonenal (4-HNE), a marker of lipid peroxidation, was increased following cisplatin exposure and substantially attenuated by dendrobine (Figure 2F). Collectively, these findings indicate that dendrobine reduces the cisplatin-induced elevation in serum Fe^2+^ levels and renal lipid peroxidation while preserving ferroptosis-associated antioxidant defenses in vivo.

### 3.3. Dendrobine Attenuates Renal Injury in Ischemia–Reperfusion-Induced AKI

**Figure 3:**
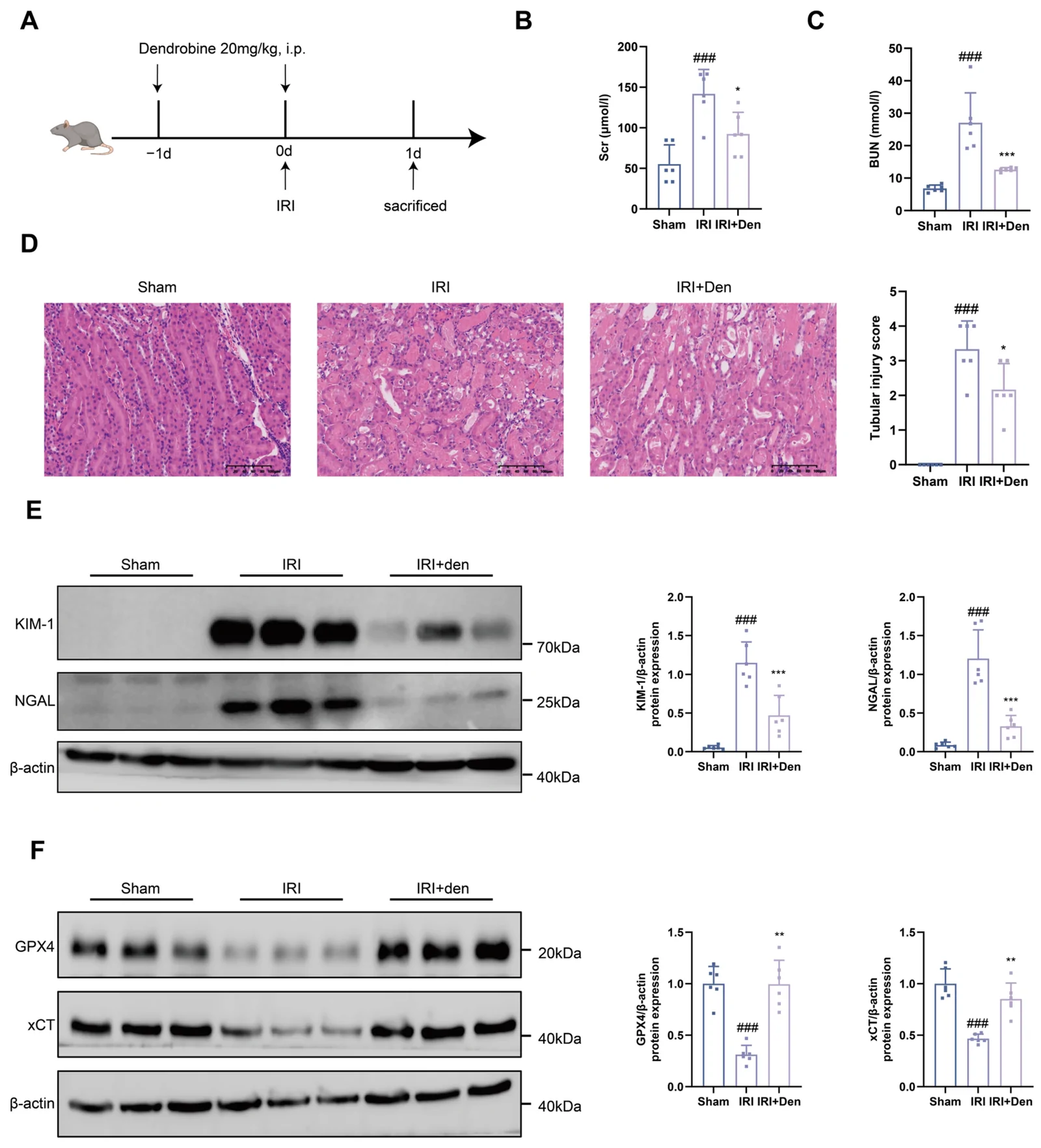
Dendrobine alleviates renal dysfunction and pathological injury in mice with renal IRI. (**A**) Schematic diagram of the experimental design. (**B**) Scr and (**C**) BUN levels. (**D**) Representative images of H&E-stained kidney sections (scale bar = 100 μm), with corresponding renal injury scores. (**E**) Western blot analysis and quantitative results of KIM-1 and NGAL protein expression. (**F**) Western blot analysis and quantitative results of GPX4 and xCT protein expression. ^###^ *p* < 0.001 vs. Sham group; * *p* < 0.05, ** *p* < 0.01, *** *p* < 0.001 vs. IRI group; n = 6.

To determine whether the renoprotective effects of dendrobine extend beyond nephrotoxic AKI, we evaluated dendrobine in a renal I/R-induced AKI model (Figure 3A). Renal I/R markedly increased serum creatinine and blood urea nitrogen levels, whereas dendrobine significantly improved both indices of renal function (Figure 3B,C). Histological examination further showed that dendrobine alleviated I/R-induced tubular damage and reduced the tubular injury score (Figure 3D). Consistently, dendrobine decreased the renal expression of the tubular injury markers KIM-1 and NGAL (Figure 3E). Moreover, the I/R-induced reductions in GPX4 and xCT expression were significantly reversed by dendrobine treatment (Figure 3F). Collectively, these findings provide supportive evidence of dendrobine-mediated renoprotection in ischemic AKI, accompanied by preservation of renal GPX4 and xCT expression.

### 3.4. Dendrobine Attenuates Ferroptosis-Associated Injury in Renal Tubular Epithelial Cells

**Figure 4:**
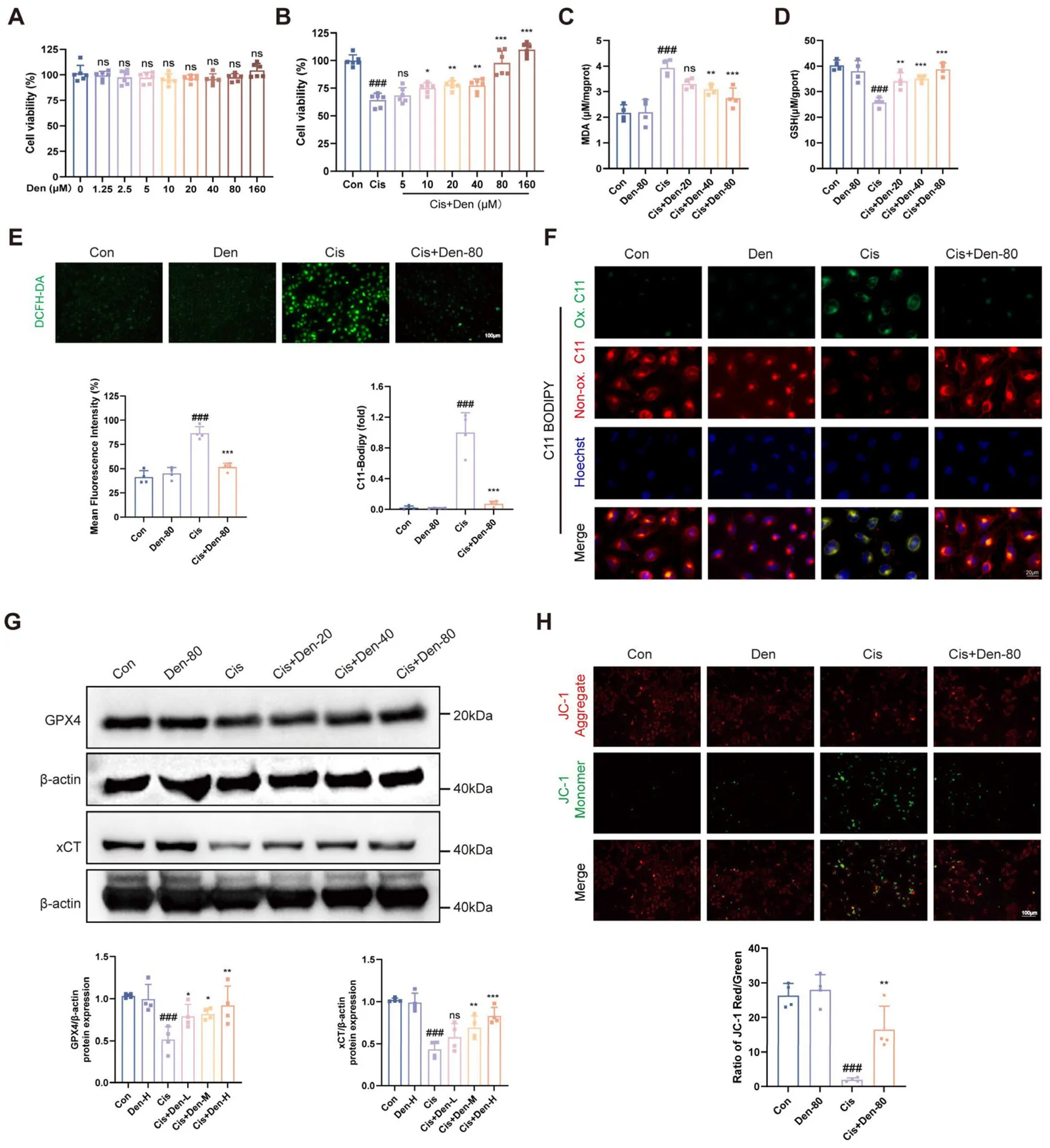
Dendrobine attenuates oxidative stress and ferroptosis-associated injury in HK-2 cells. (**A**) HK-2 cells were treated with dendrobine alone (1.25–160 μM) for 24 h, and cell viability was measured using the CCK-8 assay (n = 6). (**B**) HK-2 cells were pretreated with dendrobine (5–160 μM) for 1 h and then exposed to cisplatin (30 μM) for 24 h; cell viability was measured using the CCK-8 assay (n = 6 per group). (**C**,**D**) Intracellular MDA and GSH levels (n = 4). (**E**) Representative images and quantitative analysis of intracellular ROS generation (n = 4; scale bar, 100 μm). (**F**) Representative BODIPY 581/591 C11 fluorescence images and quantitative analysis of lipid peroxidation (n = 4; scale bar, 20 μm). (**G**) Representative immunoblots and quantitative analysis of GPX4 and xCT protein levels (n = 4). (**H**) Representative JC-1 fluorescence images and quantitative analysis of mitochondrial membrane potential (n = 4; scale bar, 100 μm). Data are presented as mean ± SD. ^###^ *p* < 0.001 versus the Con group; * *p* < 0.05, ** *p* < 0.01, and *** *p* < 0.001 versus the Cis group; ns, not significant.

To determine whether dendrobine attenuates ferroptosis-associated injury in renal tubular epithelial cells, we first evaluated its cytotoxicity and cytoprotective effects in HK-2 cells. CCK-8 assays showed that dendrobine (1.25–160 μM) had no significant effect on HK-2 cell viability, indicating good cellular tolerability within this concentration range (Figure 4A). In the cisplatin-induced injury model, dendrobine dose-dependently restored cell viability and significantly alleviated cisplatin-induced cytotoxicity (Figure 4B). We next examined biochemical changes associated with ferroptosis. Compared with the control group, cisplatin treatment markedly increased intracellular malondialdehyde (MDA) levels while reducing glutathione (GSH) content, indicative of enhanced lipid peroxidation and impaired antioxidant capacity. These alterations were significantly reversed by dendrobine treatment (Figure 4C,D). Consistently, DCFH-DA fluorescence demonstrated that dendrobine markedly attenuated cisplatin-induced intracellular ROS accumulation (Figure 4E). Likewise, BODIPY™ 581/591 C11 staining revealed a pronounced oxidation-dependent fluorescence shift from red to green following cisplatin exposure, indicating increased lipid peroxidation, which was substantially suppressed by dendrobine treatment (Figure 4F). Western blot analysis further showed that cisplatin markedly reduced the expression of the ferroptosis-related antioxidant proteins GPX4 and xCT (SLC7A11), whereas dendrobine significantly restored their expression (Figure 4G). Because mitochondrial dysfunction can accompany ferroptotic stress, mitochondrial membrane potential was further assessed using JC-1 staining. Cisplatin exposure resulted in a marked loss of mitochondrial membrane potential in HK-2 cells, whereas dendrobine substantially restored mitochondrial membrane potential, indicating an improvement in mitochondrial function (Figure 4H). Collectively, these findings show that dendrobine protects renal tubular epithelial cells from cisplatin-induced injury, accompanied by reduced lipid peroxidation, preserved GPX4/xCT expression, and maintained mitochondrial membrane potential, consistent with attenuation of ferroptosis-associated stress.

### 3.5. Dendrobine Protects HK-2 Cells Against Erastin-Induced Ferroptosis-Associated Injury

**Figure 5:**
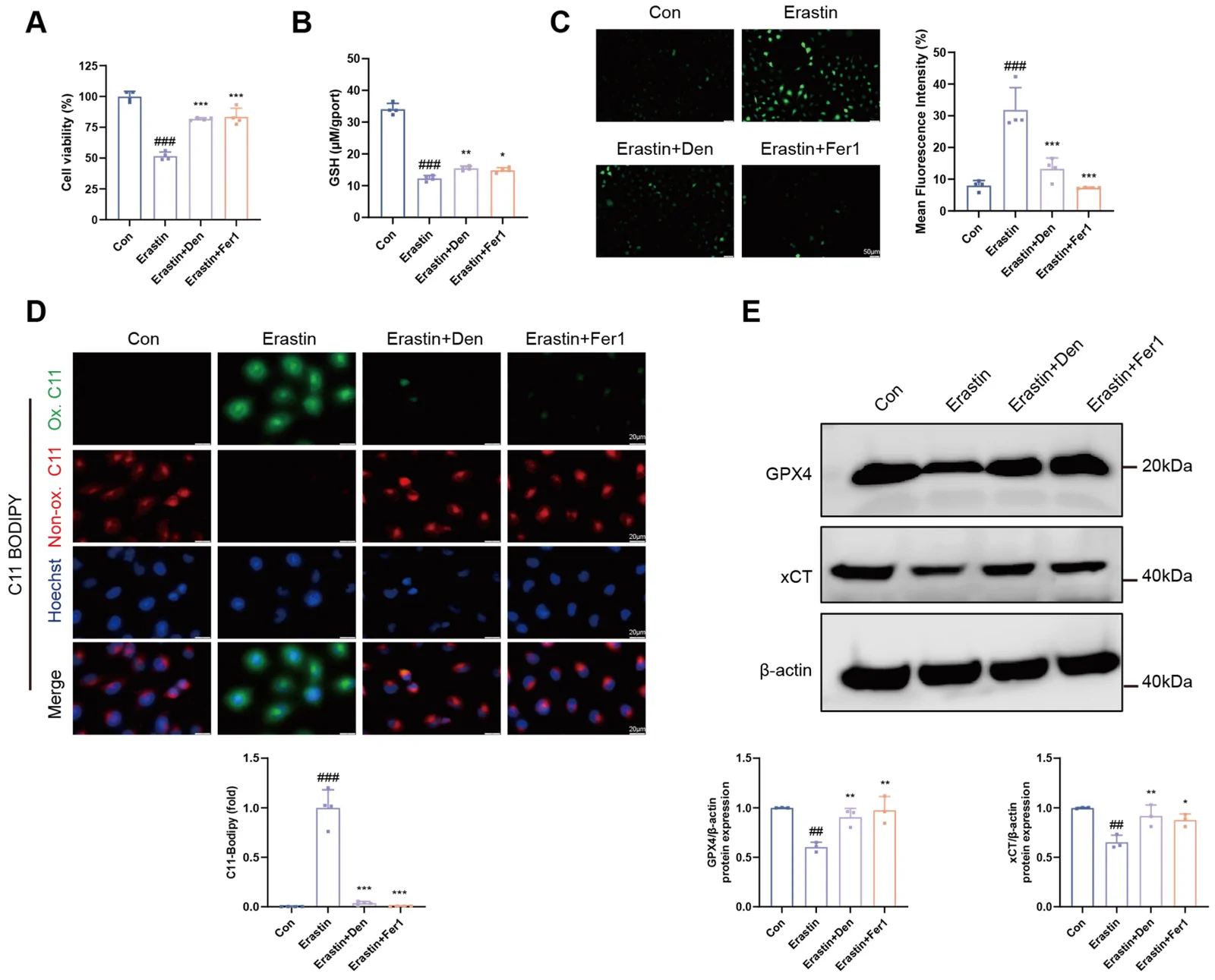
Dendrobine protects HK-2 cells against erastin-induced ferroptosis-associated injury. HK-2 cells were pretreated with dendrobine (80 μM) or ferrostatin-1 (Fer-1; 5 μM) for 1 h and subsequently exposed to erastin (5 μM) for 24 h. (**A**) Cell viability measured using the CCK-8 assay (n = 4). (**B**) Intracellular glutathione (GSH) levels (n = 4). (**C**) Representative images and quantitative analysis of intracellular ROS generation (n = 4; scale bar, 100 μm). (**D**) Representative BODIPY 581/591 C11 fluorescence images and quantitative analysis of lipid peroxidation (n = 4; scale bar, 20 μm). (**E**) Representative immunoblots and quantitative analysis of GPX4 and xCT protein levels (n = 4). Data are presented as mean ± SD. ^##^ *p* < 0.01 and ^###^ *p* < 0.001 versus the Con group; * *p* < 0.05, ** *p* < 0.01, and *** *p* < 0.001 versus the erastin group.

We next evaluated the effects of dendrobine on erastin-induced injury in HK-2 cells. As determined by the CCK-8 assay, erastin treatment significantly reduced cell viability compared with the control group, whereas dendrobine markedly restored cell viability. Notably, the cytoprotective effect of dendrobine was comparable to that of the ferroptosis inhibitor ferrostatin-1 (Fer-1) (Figure 5A). Consistent with ferroptosis-associated injury, erastin exposure caused a marked depletion of intracellular glutathione (GSH), which was effectively restored by dendrobine treatment to a level comparable to that observed with Fer-1 (Figure 5B). Furthermore, DCFH-DA staining demonstrated that erastin markedly increased intracellular ROS generation, whereas dendrobine significantly attenuated ROS accumulation (Figure 5C). Likewise, BODIPY™ 581/591 C11 staining revealed a pronounced oxidation-dependent fluorescence shift from red to green following erastin treatment, indicating enhanced lipid peroxidation, which was substantially suppressed by dendrobine (Figure 5D). Western blot analysis further demonstrated that erastin markedly reduced the expression of the ferroptosis-related antioxidant proteins GPX4 and xCT (SLC7A11), whereas dendrobine significantly restored their expression (Figure 5E). Collectively, these findings further support the notion that the cytoprotective effect of dendrobine is associated with attenuation of ferroptosis-associated stress.

### 3.6. NRF2 Inhibition Attenuates Dendrobine-Mediated Protection Against Ferroptosis-Associated Stress

**Figure 6:**
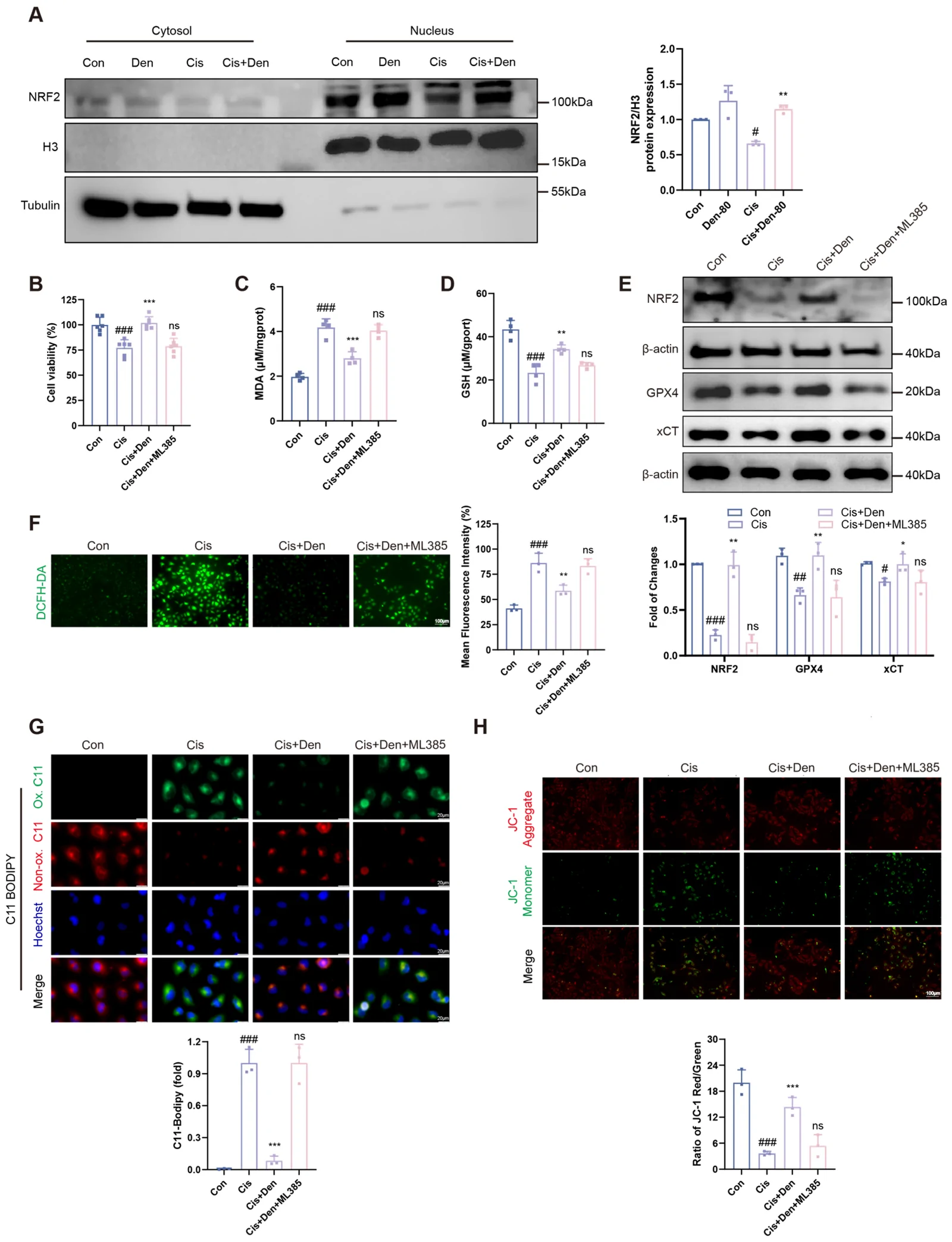
NRF2 inhibition attenuates dendrobine-mediated antioxidant protection against ferroptosis-associated stress. HK-2 cells were pretreated with ML385 (5 μM) for 1 h, followed by dendrobine (80 μM) treatment for 1 h and cisplatin (30 μM) exposure for 24 h. (**A**) Representative immunoblots and quantitative analysis of NRF2 in cytoplasmic and nuclear fractions. Histone H3 and tubulin were used as nuclear and cytoplasmic loading controls, respectively (n = 3). (**B**) Cell viability was determined by CCK-8 assay (n = 6). (**C**,**D**) Intracellular MDA and GSH levels (n = 4). (**E**) Representative immunoblots and quantitative analysis of NRF2, GPX4, and xCT protein levels (n = 3). (**F**) Representative images and quantitative analysis of ROS in HK-2 cells (n = 3, scale bar = 100 μm). (**G**) Representative BODIPY™ 581/591 C11 fluorescence images and quantitative analysis of lipid peroxidation (n = 3, scale bar = 20 μm). (**H**) Representative JC-1 fluorescence images and quantitative analysis of mitochondrial membrane potential (red/green fluorescence ratio) (n = 3, scale bar = 100 μm). ^#^ *p* < 0.05, ^##^ *p* < 0.01, ^###^ *p* < 0.001 vs. Con group; * *p* < 0.05, ** *p* < 0.01, *** *p* < 0.001 vs. Cis group, and ns, not significant vs. the Cis group.

To assess the contribution of NRF2 to dendrobine-mediated protection against ferroptosis-associated stress, we first examined NRF2 activation. Western blot analysis of nuclear and cytoplasmic fractions showed that cisplatin reduced nuclear NRF2 accumulation, whereas dendrobine promoted NRF2 nuclear translocation (Figure 6A). We then used ML385 to pharmacologically inhibit NRF2 signaling. ML385 attenuated dendrobine-mediated protection, as evidenced by reduced cell viability, increased MDA levels, and decreased GSH content (Figure 6B–D). ML385 also diminished dendrobine-induced NRF2 activation and restoration of GPX4 and xCT expression (Figure 6E) while increasing intracellular ROS and lipid peroxidation despite dendrobine treatment (Figure 6F,G). Preservation of mitochondrial membrane potential by dendrobine was likewise attenuated (Figure 6H). These pharmacological data support an important contribution of NRF2 signaling to dendrobine-mediated cytoprotection and attenuation of ferroptosis-associated oxidative stress.

### 3.7. Dendrobine Engages Cellular KEAP1 and Inhibits NRF2 Ubiquitination

**Figure 7:**
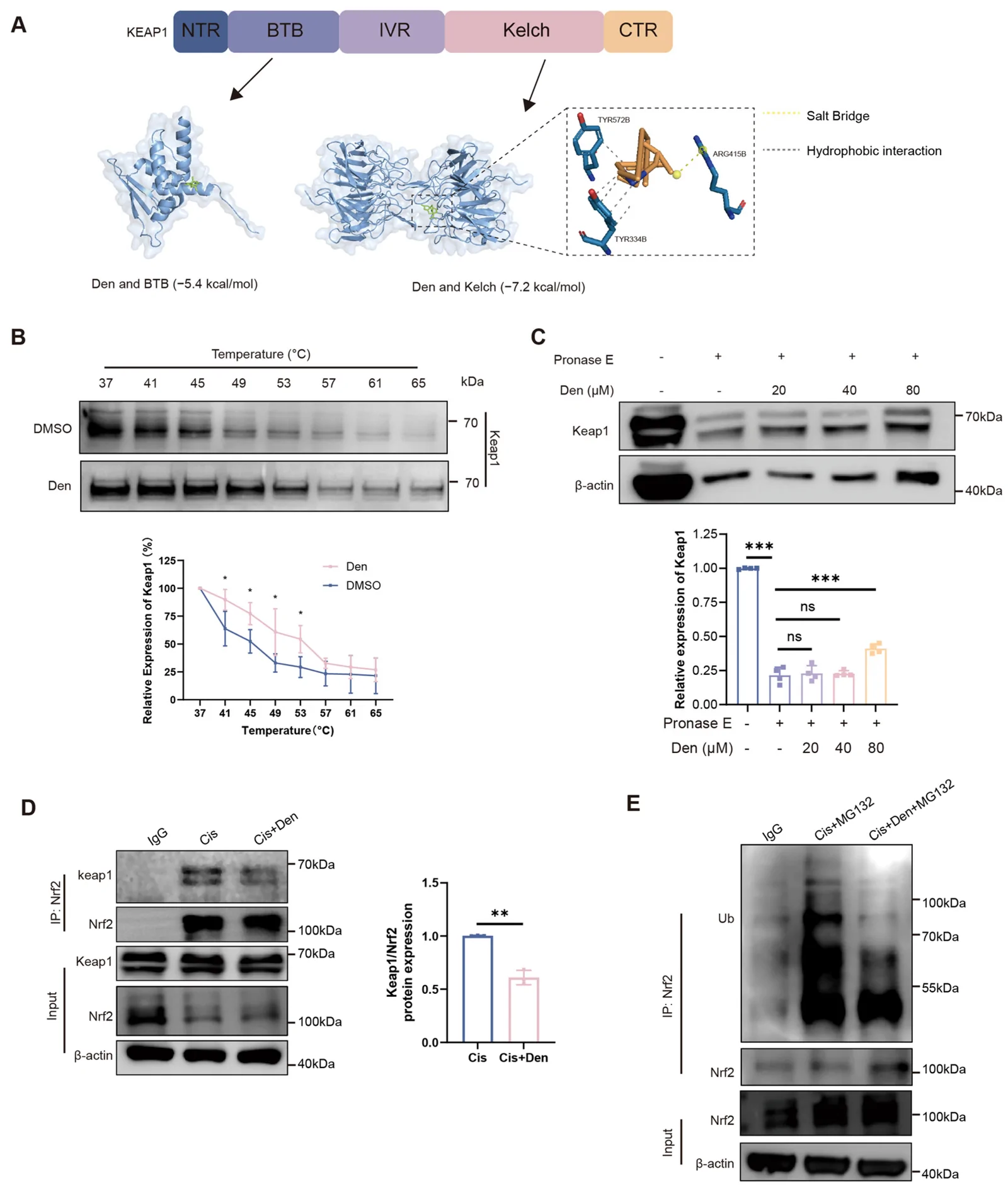
Dendrobine engages cellular KEAP1 and inhibits NRF2 ubiquitination. (**A**) Molecular docking pose of dendrobine within the Kelch domain of KEAP1. (**B**) CETSA–Western blot analysis of KEAP1 thermal stability following dendrobine treatment across a temperature gradient (n = 4). (**C**) DARTS–Western blot analysis of KEAP1 resistance to pronase digestion in the presence of dendrobine (n = 4). (**D**) Co-immunoprecipitation and quantitative analysis of the KEAP1–NRF2 interaction in HK-2 cells (n = 3). (**E**) NRF2 ubiquitination analyzed by immunoprecipitation followed by immunoblotting in HK-2 cells treated with dendrobine and cisplatin; MG132 (10 μM) was added for 6 h before collection (n = 3). * *p* < 0.05, ** *p* < 0.01, *** *p* < 0.001; ns, not significant.

To determine how dendrobine activates NRF2, we examined whether it engages cellular KEAP1. Molecular docking predicted a pose within the KEAP1 Kelch domain and identified Arg415 as a candidate interaction determinant (Figure 7A). CETSA showed that dendrobine increased the thermal stability of KEAP1 in HK-2 cells (Figure 7B), and DARTS showed reduced pronase-mediated degradation of KEAP1 in the presence of dendrobine (Figure 7C). Together, these orthogonal assays support cellular engagement of KEAP1 but do not define binding affinity or the precise interaction mode. Co-immunoprecipitation further showed that dendrobine reduced the KEAP1–NRF2 interaction in cisplatin-treated HK-2 cells (Figure 7D). Consistently, dendrobine decreased NRF2 ubiquitination (Figure 7E), supporting stabilization of NRF2 through attenuation of KEAP1-mediated ubiquitination. Thus, dendrobine engages cellular KEAP1, alters the KEAP1–NRF2 interaction, and promotes NRF2 activation.

### 3.8. Arg415 Contributes to Dendrobine-Mediated Cellular KEAP1 Engagement and Protection Against Ferroptosis-Associated Stress

**Figure 8:**
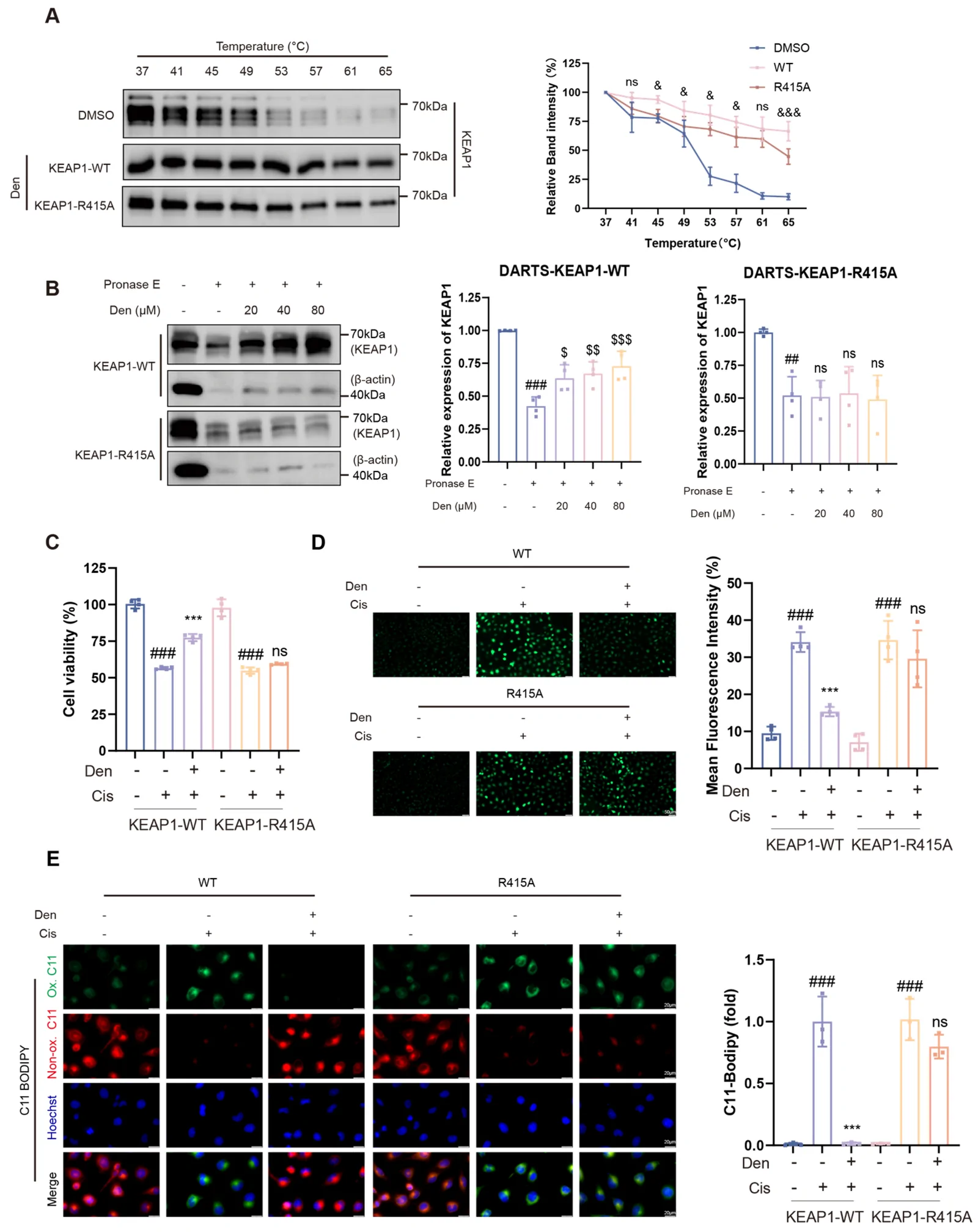
Arg415 contributes to dendrobine-mediated KEAP1 engagement and protection against ferroptosis-associated stress. (**A**) CETSA analysis of KEAP1 thermal stability in WT and R415A mutant cells treated with dendrobine (n = 4). (**B**) DARTS analysis of KEAP1 susceptibility to Pronase E digestion in WT and R415A mutant cells following dendrobine treatment (n = 4). (**C**) Cell viability determined by CCK-8 assay (n = 4). (**D**) Representative images and quantitative analysis of intracellular ROS levels (n = 4, scale bar = 100 μm). (**E**) Representative images and quantitative analysis of lipid peroxidation detected by C11-BODIPY staining (n = 4, scale bar = 20 μm). ^##^ *p* < 0.01, ^###^ *p* < 0.001 vs. Con group; *** *p* < 0.001 vs. Cis group; & *p* < 0.05, &&& *p* < 0.001 vs. WT group; $ *p* < 0.05, $$ *p* < 0.01, $$$ *p* <0.001 vs. Pronase E group and ns: not significant.

To test whether Arg415 contributes to the cellular response to dendrobine, we compared wild-type KEAP1 with the R415A mutant. Dendrobine increased the thermal stability of wild-type KEAP1, whereas this change was attenuated by the R415A substitution (Figure 8A). Similarly, dendrobine protected wild-type KEAP1 from pronase-mediated degradation to a greater extent than KEAP1-R415A (Figure 8B), supporting an Arg415-dependent effect on cellular KEAP1 engagement. Functionally, dendrobine restored cell viability in cells expressing wild-type KEAP1, whereas this protection was attenuated in cells expressing KEAP1-R415A (Figure 8C). The R415A substitution also diminished dendrobine-mediated reductions in intracellular ROS and lipid peroxidation (Figure 8D,E). Together, these findings show that the cellular responses to dendrobine are Arg415-sensitive and extend the functional relevance of the previously reported dendrobine–KEAP1/Arg415 interaction to renal tubular protection against ferroptosis-associated stress.

### 3.9. Arg415 Contributes to Dendrobine-Mediated Modulation of KEAP1–NRF2 Signaling

**Figure 9:**
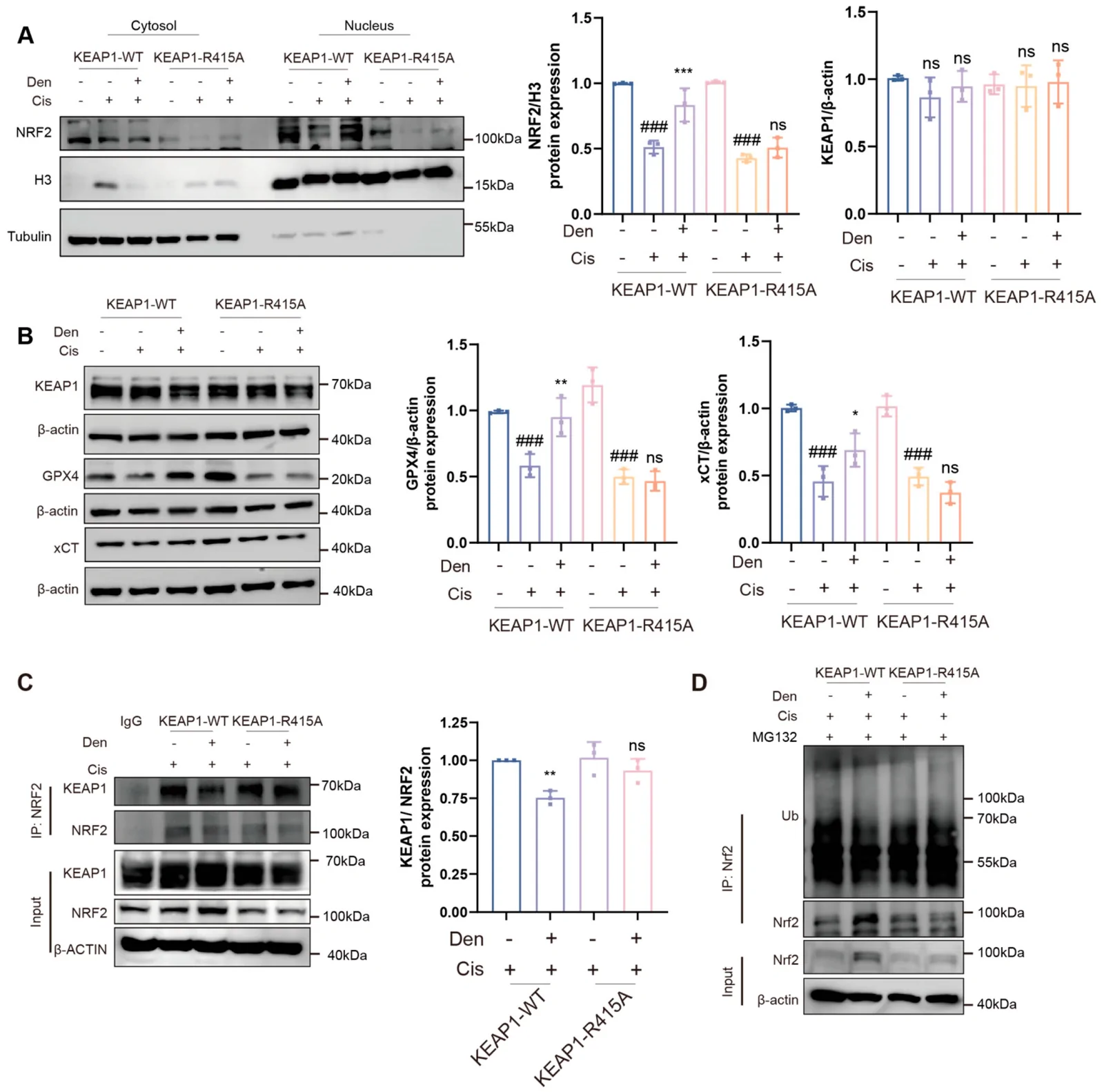
Arg415 contributes to dendrobine-mediated activation of KEAP1–NRF2 signaling. (**A**) Representative immunoblots and quantitative analysis of cytoplasmic and nuclear NRF2 levels in HK-2 cells expressing wild-type KEAP1 (KEAP1-WT) or KEAP1-R415A following the indicated treatments (n = 4). (**B**) Representative immunoblots and quantitative analysis of KEAP1, GPX4 and xCT protein levels (n = 4). (**C**) Representative co-immunoprecipitation results and quantitative analysis of the KEAP1–NRF2 interaction (n = 3). (**D**) HK-2 cells expressing KEAP1-WT or KEAP1-R415A were treated with dendrobine and cisplatin as indicated. MG132 (10 μM) was added 6 h before cell collection, and NRF2 ubiquitination was assessed by immunoprecipitation followed by immunoblotting (n = 3). Data are presented as mean ± SD. ^###^ *p* < 0.001 versus the Con group; * *p* < 0.05, ** *p* < 0.01, and *** *p* < 0.001 versus the Cis group; ns, not significant.

We next examined whether KEAP1-R415A altered dendrobine-mediated regulation of the KEAP1–NRF2 pathway. Dendrobine promoted nuclear accumulation of NRF2 in cells expressing wild-type KEAP1, whereas this response was attenuated in cells expressing KEAP1-R415A (Figure 9A). The R415A substitution similarly diminished dendrobine-mediated restoration of GPX4 and xCT expression. KEAP1 protein levels did not differ significantly among the experimental groups, suggesting that these effects were not attributable to changes in KEAP1 abundance (Figure 9B). Co-immunoprecipitation showed that dendrobine reduced the KEAP1–NRF2 interaction in cells expressing wild-type KEAP1, whereas this effect was attenuated in the R415A background (Figure 9C). Dendrobine also reduced NRF2 ubiquitination in the presence of wild-type KEAP1, but this response was diminished after Arg415 substitution (Figure 9D). Together, these results support a functional contribution of Arg415 to dendrobine-mediated regulation of NRF2 stability and antioxidant signaling.

## 4. Discussion

In the present study, dendrobine substantially attenuated cisplatin-induced AKI, improving renal function, tubular morphology, and injury-marker expression. In renal tubular cells, dendrobine reduced cisplatin- and erastin-associated lipid peroxidation and preserved the GSH–GPX4/xCT antioxidant defense system. Our mechanistic experiments showed cellular KEAP1 engagement, reduced KEAP1–NRF2 association and NRF2 ubiquitination, and increased nuclear accumulation of NRF2; pharmacological NRF2 inhibition attenuated dendrobine-mediated cytoprotection. Together, these findings connect the dendrobine–KEAP1–NRF2 pathway to renal tubular protection and ferroptosis-associated stress in cisplatin-induced injury. We also observed improved renal outcomes in an I/R model, providing additional evidence of renoprotective activity.

Ferroptosis is increasingly recognized as an important component of renal tubular injury in nephrotoxic and ischemic AKI [37,38]. Renal tubular epithelial cells are particularly susceptible to disturbances in iron metabolism, glutathione depletion, and phospholipid peroxidation because of their high metabolic activity and dependence on mitochondrial function. Although direct inhibition of iron accumulation or lipid peroxidation can alleviate experimental AKI, reinforcement of endogenous antioxidant defenses may provide broader protection against the interacting forms of oxidative damage that occur during tubular injury. In the present study, dendrobine restored GSH availability, preserved GPX4 and SLC7A11/xCT expression, and reduced lipid peroxidation in cisplatin-challenged kidneys and tubular epithelial cells. Its protective activity against erastin-induced injury further supports an effect on ferroptosis-related redox vulnerability rather than an action restricted to the direct toxic effects of cisplatin. In the renal I/R model, dendrobine also improved renal function and tubular injury and preserved GPX4 and xCT expression. These findings provide supportive evidence of renoprotection, although the involvement of KEAP1–NRF2 signaling and ferroptosis in this model requires further investigation.

NRF2 is a central regulator of cellular adaptation to oxidative stress and an important determinant of ferroptosis sensitivity [14,39]. By inducing genes involved in glutathione synthesis, cystine uptake, detoxification, and lipid peroxide removal, NRF2 coordinates multiple components of the antioxidant response rather than regulating a single ferroptosis effector. Previous studies have implicated NRF2 activation in the antioxidant and cytoprotective effects of dendrobine [26,28,40]. Xiang et al. reported direct dendrobine–KEAP1 binding, disruption of the KEAP1–NRF2 interaction, and the involvement of Arg415 in a diabetic cardiomyopathy model [27]. Our independently initiated study examined dendrobine in the distinct setting of cisplatin-induced AKI and renal tubular injury. In this setting, dendrobine improved renal outcomes, preserved the SLC7A11/xCT–GSH–GPX4 antioxidant defense system, and reduced lipid peroxidation, consistent with attenuation of ferroptosis-associated stress.

Our mechanistic analyses further support the involvement of the KEAP1–NRF2 pathway in the renoprotective effects of dendrobine. Molecular docking suggested a potential interaction within the KEAP1 Kelch domain, whereas CETSA and DARTS supported cellular target engagement. Co-immunoprecipitation and ubiquitination analyses showed that dendrobine reduced the KEAP1–NRF2 association and NRF2 ubiquitination. Moreover, the R415A substitution attenuated dendrobine-induced NRF2 stabilization, restoration of GPX4 and SLC7A11/xCT expression, and protection against ferroptosis-associated stress, supporting the functional contribution of this residue to pathway regulation. Nevertheless, because the present assays do not determine binding affinity or define the precise interaction mode, additional quantitative biophysical and structural studies are warranted.

The present findings may have implications for the development of redox-directed interventions for AKI. Pharmacological modulation of KEAP1–NRF2 signaling may reinforce several endogenous antioxidant processes simultaneously. Nevertheless, several limitations should be considered. The pharmacokinetic properties, renal distribution, bioavailability, and therapeutic window of dendrobine under the dosing conditions used here remain to be established. Because cisplatin is an anticancer agent, it will also be necessary to determine whether dendrobine reduces nephrotoxicity without compromising its antitumor activity. Our biochemical findings and results in erastin-challenged cells support attenuation of ferroptosis-associated stress; however, whether this process is causally required for dendrobine-mediated protection in the cisplatin model remains to be established using ferroptosis-specific rescue or genetic approaches. The Fe^2+^ measurements reflect circulating changes rather than renal iron content, and therefore whether dendrobine affects renal iron accumulation remains unresolved. Although the present mutational analyses support a functional contribution of KEAP1 Arg415, renal tubule-specific genetic manipulation of NRF2 or KEAP1 would provide stronger evidence for the causal involvement of this pathway in vivo. The contributions of other renal cell populations, regulated cell-death pathways, and inflammatory processes also warrant further investigation. Finally, the I/R findings provide supportive evidence of renoprotective activity, but the limited mechanistic evaluation in this model does not establish whether KEAP1–NRF2 signaling or ferroptosis-associated mechanisms contribute to dendrobine-mediated protection in ischemic AKI or other AKI etiologies.

## 5. Conclusions

Our findings identify dendrobine as a potential renoprotective agent against cisplatin-induced AKI. Dendrobine alleviates renal tubular injury and oxidative lipid damage while preserving the SLC7A11/xCT–GSH–GPX4 antioxidant defense system. Mechanistically, dendrobine engages KEAP1 and promotes NRF2 stabilization and antioxidant activation, with KEAP1 Arg415 contributing to these responses in renal tubular cells. These effects are accompanied by attenuation of ferroptosis-associated stress, supporting KEAP1–NRF2 signaling as an important component of dendrobine-mediated tubular protection. Dendrobine also confers supportive renoprotection in renal I/R injury, although the underlying mechanisms in this setting remain to be determined. Collectively, these findings provide a rationale for further investigation of dendrobine as a potential therapeutic strategy for AKI and ferroptosis-associated renal injury.

## Data Availability

The original contributions presented in this study are included in the article/Supplementary Material. Further inquiries can be directed to the corresponding authors.

## Supplementary Materials

The following supporting information can be downloaded at https://www.mdpi.com/article/10.3390/antiox15101337/s1, Figure S1: Dendrobine suppresses tubular inflammation and apoptosis in cisplatin-induced AKI; Table S1: Primary and Secondary Antibodies Used in This Study.

## Abbreviations

The following abbreviations are used in this manuscript:

AKI	acute kidney injury
BUN	blood urea nitrogen
CCK-8	Cell Counting Kit-8
CETSA	cellular thermal shift assay
Co-IP	co-immunoprecipitation
DARTS	drug affinity responsive target stability
GSH	glutathione
IL-1β	interleukin-1 beta
IL-6	interleukin-6
I/R	ischemia–reperfusion
IRI	ischemia–reperfusion injury
KEAP1	Kelch-like ECH-associated protein 1
KIM-1	kidney injury molecule-1
MDA	malondialdehyde
NGAL	neutrophil gelatinase-associated lipocalin
NRF2	nuclear factor erythroid 2-related factor 2
ROS	reactive oxygen species
Scr	serum creatinine
TNF-α	tumor necrosis factor alpha
