# Supplementary material for: Dendrobine Attenuates Cisplatin-Induced Acute Kidney Injury with KEAP1–NRF2 Activation and Reduced Ferroptosis-Associated Stress
Source: Antioxidants (Basel). 2026 Oct 16;15(10):1337. doi: 10.3390/antiox15101337 (PMC13701420; doi:10.3390/antiox15101337)
Supplement: Supplementary file 1 [file antioxidants-15-01337-s001.zip › antioxidants-4580371-supplementary.pdf]

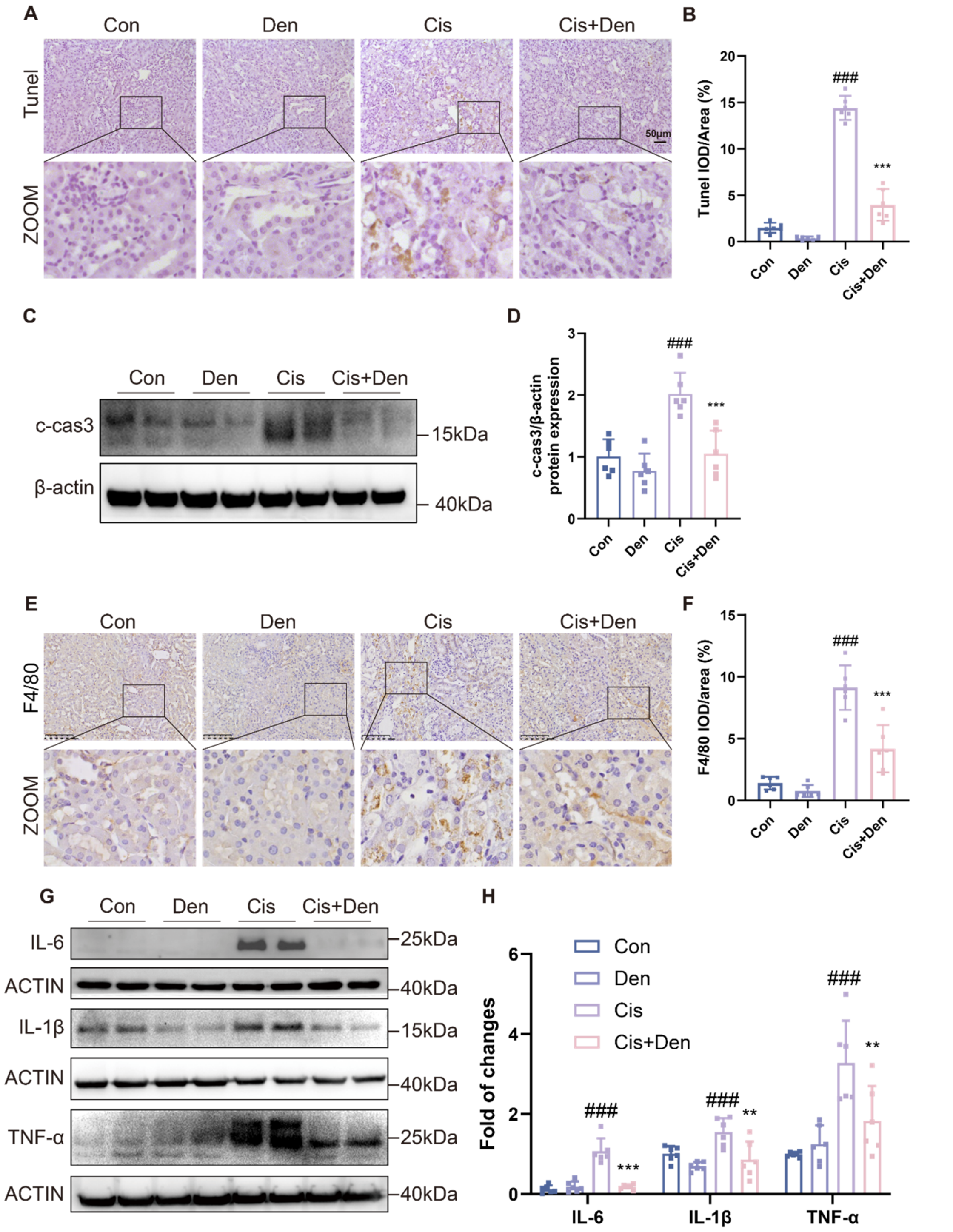

Figure S1. Dendrobine suppresses tubular inflammation and apoptosis in cisplatin-induced AKI. (A–B) Representative TUNEL staining and quantification of TUNEL-positive cells. Scale bar, 50 μm. (C–D) Representative immunoblots and quantitative analysis of cleaved caspase-3 (c-CASP3). (E–F) Representative immunohistochemical staining and quantification of F4/80-positive macrophages. Scale bar, 100 μm. (G–H) Representative immunoblots and quantitative analysis of IL-6, IL-1β, and TNF-α. Protein

levels were normalized to β-actin and expressed relative to the corresponding control group. Data are presented as mean ± SD. Each dot represents an individual mouse. $^{###}P < 0.001$ versus the Con group; $^{**}P < 0.01$ and $^{***}P < 0.001$ versus the Cis group. n = 6 mice per group.

Table S1. Primary and Secondary Antibodies Used in This Study

Antibody	*Company*	*Category*	*Application*	*Dilution*
GPX4	Abmart	T56959	WB; IHC	1:2000; 1:200
SLC7A11/xCT	Abmart	TD12509	WB; IHC	1:1000; 1:200
Rabbit IgG	Abmart	B30011	WB	1:100
NRF2	Abmart	T55136	WB	1:1000
IL-1β	Abclonal	A16288	WB	1:500
IL-6	Abclonal	A0286	WB	1:500
TNF-α	Abcam	ab183218	WB	1:1000
F4/80	Huabio	HA721520	IHC	1:200
Ubiquitin	Huabio	ET1609-21	WB	1:2000
4-HNE	R&D system	MAB3249	IHC	1:500
KIM-1	R&D system	AF1817	WB	1:1000
NGAL	R&D system	MLCN20	WB	1:1000
Cleaved-caspase3	Cell Signaling Technology	9664	WB	1:1000
KEAP1	Proteintech	80744-1-RR	WB	1:2000
H3	Servicebio	GB15102	WB	1:2000
β-actin	Proteintech	HRP-66009	WB	1:4000
β-Tubulin	Proteintech	HRP-66240	WB	1:4000
HRP-conjugated mouse	Abclonal	AS003	WB	1:4000
HRP-conjugated rabbit	Abclonal	AS014	WB	1:4000
HRP-conjugated goat	Proteintech	SA00001-4	WB	1:4000
Svision polymer secondary antibody	Servicebio	G1313	IHC	/
